# A case with burning mouth syndrome followed by dementia with Lewy bodies: a case report

**DOI:** 10.3389/fpsyt.2023.1329171

**Published:** 2024-01-08

**Authors:** Motoko Watanabe, Wataru Araki, Chihiro Takao, Chizuko Maeda, Risa Tominaga, Yasuyuki Kimura, Gayatri Nayanar, Trang Thi Huyen Tu, Takashi Asada, Akira Toyofuku

**Affiliations:** ^1^Department of Psychosomatic Dentistry, Graduate School of Medical and Dental Sciences, Tokyo Medical and Dental University, Tokyo, Japan; ^2^Department of Neurology and Neurological Science, Graduate School of Medical and Dental Sciences, Tokyo Medical and Dental University, Tokyo, Japan; ^3^Memory Clinic Ochanomizu, Tokyo, Japan; ^4^Department of Basic Dental Sciences, Faculty of Odonto-Stomatology, University of Medicine and Pharmacy at Ho Chi Minh City, Ho Chi Minh City, Vietnam

**Keywords:** burning mouth syndrome, dementia, dementia with Lewy bodies, elderly patient, cognitive decline, hallucination, Capgras syndrome, antidepressant

## Abstract

Burning mouth syndrome (BMS) is characterized by persistent oral burning sensations without corresponding organic findings. Dementia with Lewy bodies (DLB) is a common type of dementia and generally presents visual hallucination and parkinsonism as motor dysfunction besides cognitive decline. In this case report, we present a case in which DLB emerged during the treatment for BMS, with a relatively positive outcome for BMS. A 74 years-old female complained of burning pain in her mouth and a subsequent decrease in food intake. Following a diagnosis of BMS, pharmacotherapy was initiated. BMS was much improved with mirtazapine 15 mg and aripiprazole 1.0 mg, leading to the restoration of her food intake by day 180. However, BMS flared up again triggered by deteriorating physical condition of herself and that of her husband. With aripiprazole 1.5 mg and amitriptyline 25 mg, her BMS gradually improved by day 482. However, by day 510, an increase in anxiety was noted, accompanied by the occasionally misidentification of her husband on day 566. Her cognitive impairment and disorientation were also reported by her husband on the day 572, she was then immediately referred to a neurologist specialized dementia and diagnosed with DLB on the day 583. Her treatment was adjusted to include the prescription of rivastigmine which was titrated up to 9.0 mg. Considering the potential impact of amitriptyline on cognitive function, it was reduced and switched to mirtazapine; however, her oral sensations slightly got worse. Following the consultation with her neurologist, amitriptyline 10 mg was reintroduced and aripiprazole was discontinued on day 755. Remarkably, BMS gradually improved without deteriorating DLB. This case indicated the reaffirmed necessity of careful interviews for changes in daily life not only with the patients but also with their families through the medical assessments. It highlights the vigilance regarding potential cognitive decline underlying or induced as an adverse event especially when treating elderly patients with BMS. While the interaction between BMS and DLB remains unclear, this case underscores the importance of prudent diagnosis and constructing collaboration with specialists in managing BMS with the early phase of DLB.

## 1 Introduction

Burning mouth syndrome (BMS) is characterized by persistent oral burning sensations without corresponding organic findings. While a potential association between cognitive decline and several chronic pain disorders has been indicated (1), no definitive connection has yet been established in individuals with burning mouth syndrome (2). Nevertheless, as the elderly population diagnosed with BMS continues to grow in the context of aging society (3), it is becoming progressively important to carefully consider the potential presence of underlying cognitive decline and the risk of adverse events associated with psychopharmacotherapy for BMS throughout the course of treatments, including the diagnosis phase, especially for elderly patients.

Dementia with Lewy bodies (DLB) is one of the most prevalent types of dementia, accounting for 10%–15% of dementia cases (4). The main features are visual hallucination and parkinsonism as motor dysfunction besides cognitive decline. Delusional oral sensations such as oral cenesthopathy has been reported as prodromal sensations for DLB (5–7). However, there is currently only a single case report in French describing development of DLB in a patient previously diagnosed with BMS. In this report, we describe a case in which DLB manifested during the treatment for BMS and remarkably, BMS showed a favorable prognosis without severe exacerbation of DLB.

## 2 Case presentation

A 74 years-old female complained of persistent burning pain in her mouth which was exacerbated on consuming foods or drinks. This discomfort had first emerged in October X-1 years without any apparent triggers and had had gradually worsened over time. By January X years, her condition had deteriorated to the point where she could tolerate specific foods such as rice, sweet breads, or pudding due to the severe pain. Her symptoms did not improve despite previous treatment with polaprezinc and antifungal drug for *Candida albicans*. Mirtazapine was prescribed by her physician considering her decreased appetite and normal blood examination results; however, she discontinued it due to drowsiness. Additionally, she could not take mirogabalin either because of dizziness. By May X years, her condition had deteriorated to extent that she required intravenous drips 4 times a week as she could only consume liquid or soft foods at room temperature. This worsening oral condition had gradually started impacting her ability to enjoy playing tennis and sewing hand-made crafts with her friends. Subsequently, she was referred to our clinic in June X years for a comprehensive evaluation and further management.

She had been diagnosed with primary Sjogren’s syndrome since January X-1 years. Since her primary symptom was only salivary dysfunction, she was prescribed sodium gualenate hydrate by her rheumatologist. Although she had no psychiatric comorbidities, her physician reported her psychological characteristics as anxiety and a pessimistic outlook. To address this, she had been prescribed alprazolam and brotizolam, to use on an as-needed basis for over 10 years.

**Figure 1:**
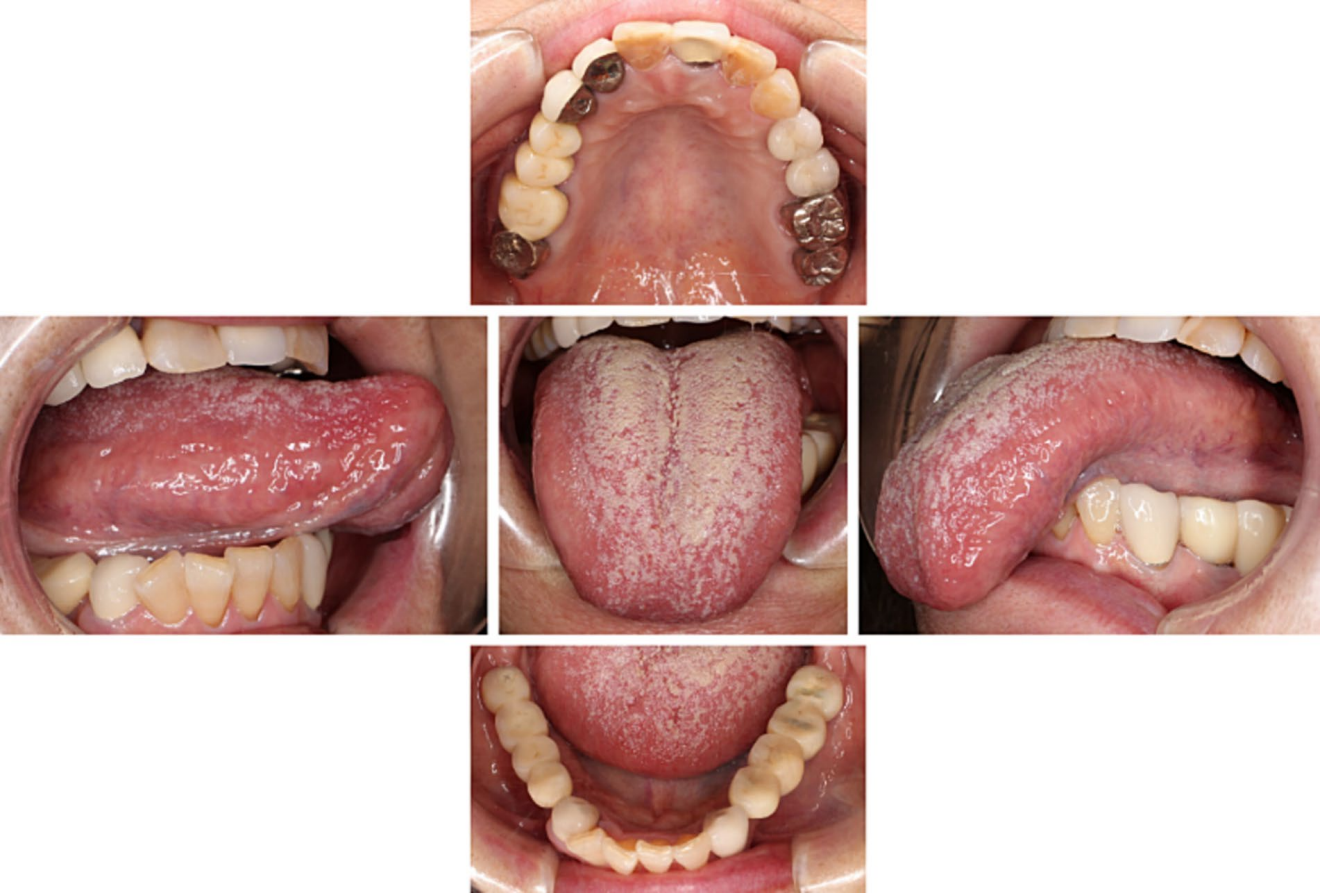
Intraoral findings. There were no abnormal findings in her oral cavity except slight tongue coating.

Accompanied by her husband, with whom she had been living alone since their children had become self-sufficient, she visited our clinic. They seemed to care for each other. During her visit, she was able to walk into our clinic unassisted, dressed neatly, and left a bright and lively impression. There were no signs of paralysis or movement disorders in the orofacial region. Although a slight tongue coating was observed, it did not cause pain upon touch (Figure 1). Decreased salivation was found but no abnormal mucosal changes were detected, including atrophy of tongue papillae. Taste dysfunction was not evident. Her self-rating depressive scale (SDS) score was 58, and visual analogue scale (VAS) which indicates pain intensity was notably high at 86/100. Furthermore, she showed a tendency for pain catastrophizing, scoring 30/52 on the pain catastrophizing scale (PCS).

**Figure 2:**
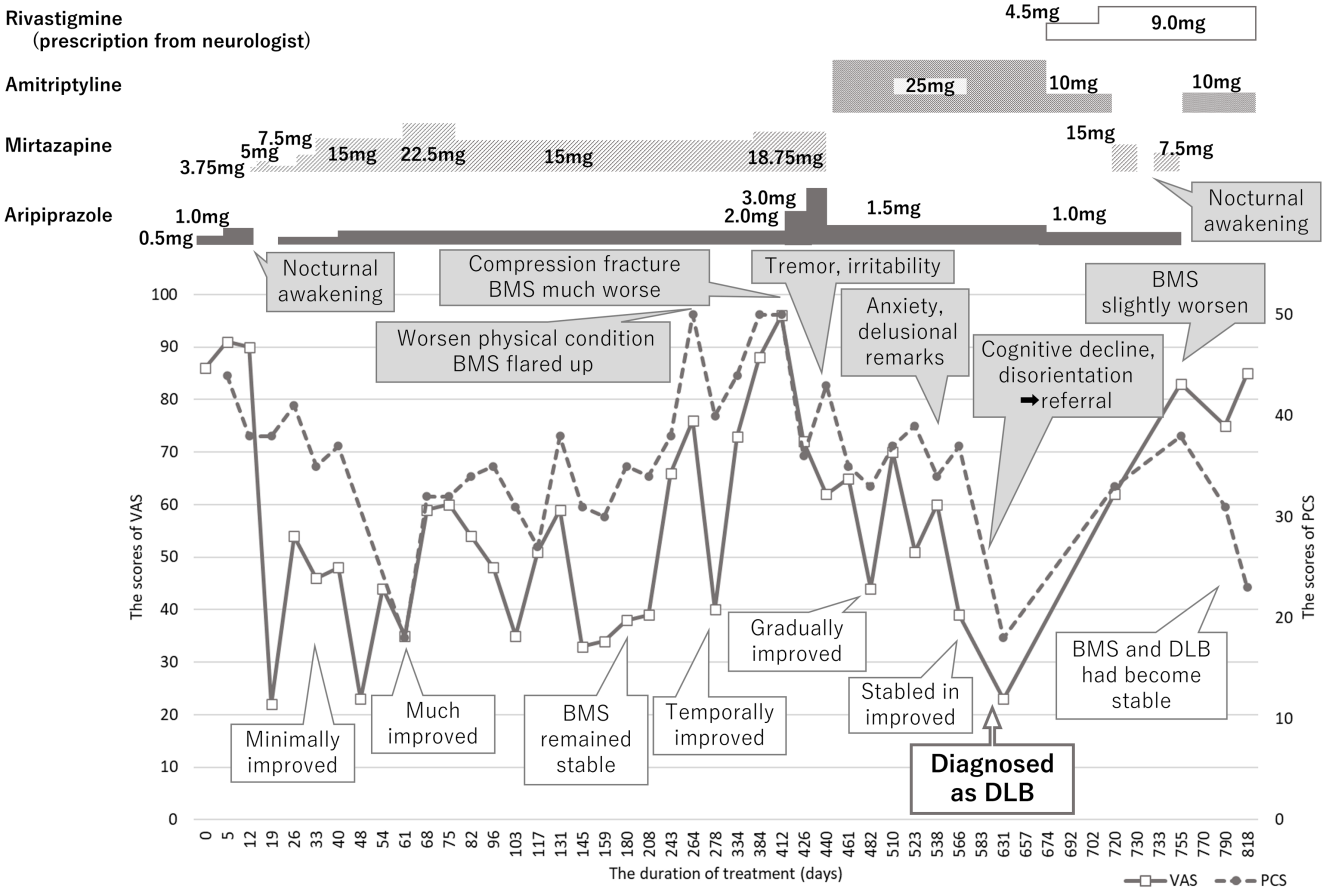
Time course of treatment. Burning mouth syndrome (BMS) was improved with mirtazapine 15 mg and aripiprazole 1.0 mg; however, it had been exacerbated relating with physical conditions of herself and her husband. While BMS had been improved, the sudden cognitive dysfunction and disorientation were reported by her husband. She was immediately referred to neurologist and diagnosed with dementia with Lewy bodies (DLB). BMS got in a good prognosis with slow progression of DLB by amitriptyline 10 mg besides rivastigmine prescribed by her neurologist. VAS, visual analogue scale, PCS, pain catastrophizing scale, BMS, burning mouth syndrome, DLB, dementia with Lewy bodies.

On being diagnosed with BMS, she was initially prescribed 0.5 mg of aripiprazole considering her previous medication sensitivity. However, she experienced nocturnal awakening when the dose was increased to 1.0 mg. Consequently, a low dose (3.75 mg) of mirtazapine was re-prescribed to promote sleep and a transition of augmenting mirtazapine with aripiprazole was made on day 19 (Figure 2). By day 33, mirtazapine 7.5 mg and aripiprazole 0.5 mg yielded a marginal reduction in BMS symptoms. A considerable improvement was noted in BMS symptoms with 15 mg of mirtazapine and 1.0 mg of aripiprazole by day 61, paralleled by a decrease in intravenous drips to twice in a week. She regained the ability to eat meat and vegetable and her condition stabilized with further improvements by day 180. Her physician observed increased food intake and reduced frequency of intravenous drips to once a week, alongside absence of spontaneous crying. The PCS scores also decreased along with decrease in VAS scores. However, BMS flared up again on day 264, triggered by her and her husband’s deteriorating physical condition and with increase of PCS (50/52). Although BMS improved temporarily with alprazolam as a rescue medication, the events such as her lumbar compression fracture and acute deteriorating of her husband’s physical condition made her more anxiety and worsened her BMS symptoms. A cessation of tennis or sewing activities with her friend was observed since she became unable to walk by herself after lumbar compression fracture, accompanied by apatite loss and about 3 to 4 kg weight decline. Her pain and anxiety symptoms fluctuated. Aripiprazole was then gradually increased up to 3.0 mg which initially improved BMS. However, since tremor and irritability occurred on day 440, aripiprazole had to be decreased to 1.5 mg and 15 mg of mirtazapine was substituted with 25 mg of amitriptyline. By day 482, her BMS gradually improved, and food intake also increased.

However, by day 510, an increase in anxiety, led to cautious approach to treatment with more frequent consultations. On day 566, her husband reported that she often made remarks such as “I wonder if my husband is going out and meeting someone else.” Her physician also reported escalated anxiety and emotional incontinence although her responses were clear and obvious recognitive decline could not be found through the medical interviews. While her BMS symptoms remained stable in the improved state, the score of SDS decreased to 36 and the frequency of intravenous drip reduced to once in 3 weeks, her husband reported cognitive impairment and disorientation. He described instances where she would say “even if you are next to me, you are not my husband but someone else with his face, and I will not sleep until real one returns” and other comments like, “this is not my house” and she could not be reassured of by going outside to confirm it was her own house. These alarming developments promoted immediately referral to a neurologist specializing in dementia on day 583.

**Figure 3:**
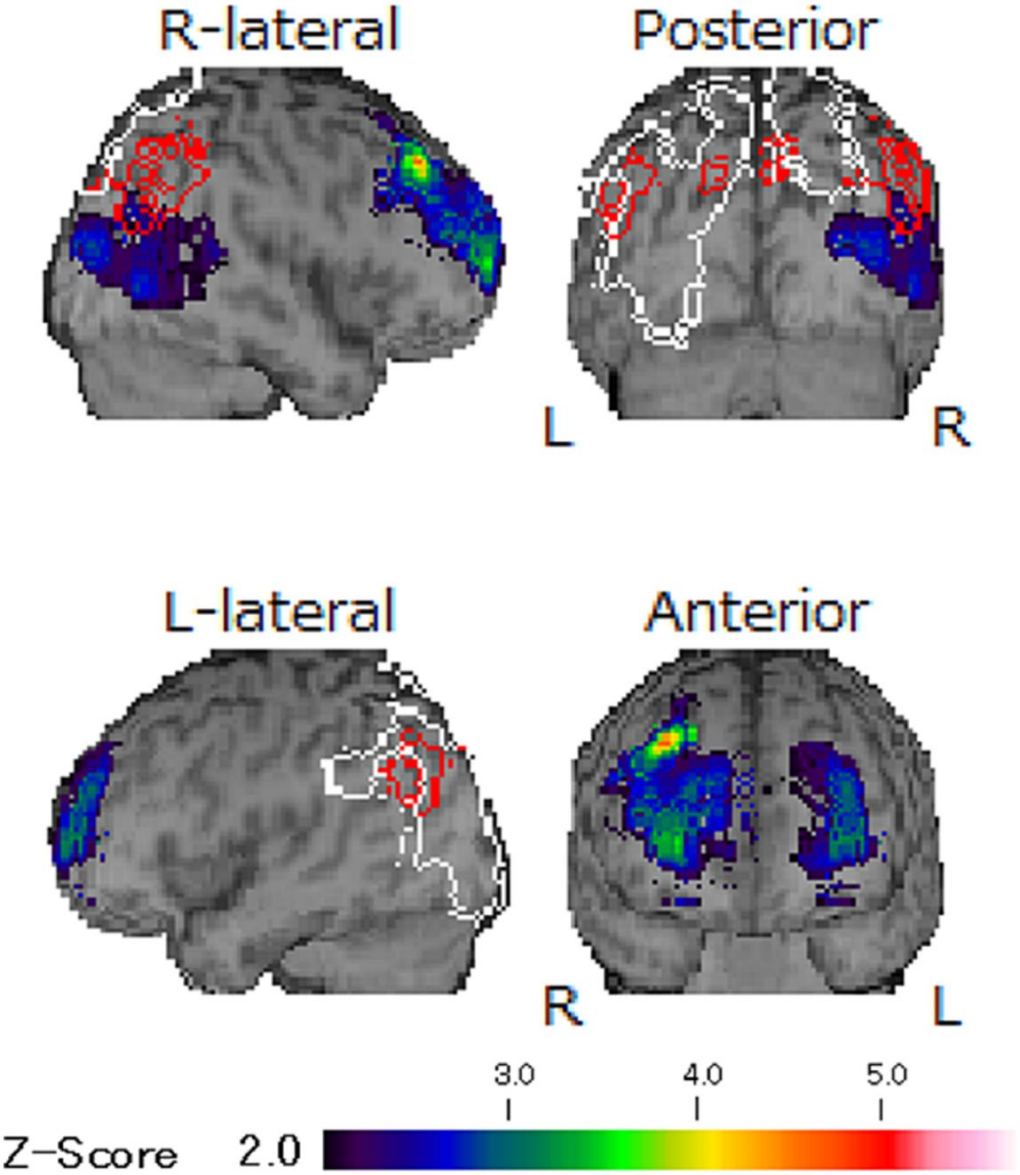
The images of single photon computed tomography. The hypoperfusion was detected only in right posterior region and any other abnormalities were not revealed according to the easy *Z* score imaging system of single photon computed tomography with 99mTc-ECD. The observed unilateral hypoperfusion is not typical finding in DLB and its degree was not enough to meet the threshold of DLB.

There were no abnormalities detected in magnetic resonance images and electro encephalography. Moreover, the hypoperfusion was detected only in the right posterior region, which was not significant enough to meet to the threshold of DLB, according to the easy *Z* score imaging system of single photon computed tomography with 99mTc-ECD (Figure 3). However, symptoms indicative of misidentification and Capgras syndrome were also observed besides decline of recognition (mini mental state examination: MMSE; 17/30, Hasegawa dementia rating scale—revised: HDSR; 15/30). Consequently, she was then diagnosed with dementia with Lewy bodies and was prescribed a 4.5 mg of rivastigmine on day 674, which was later increased to 9 mg on day 702. Considering the impact of amitriptyline on cognitive function, it was reduced and switched to mirtazapine; unfortunately, this resulted into slight worsening of her oral sensations. Since discontinuation of mirtazapine induced sleep disturbance, mirtazapine 7.5 mg and aripiprazole was prescribed again on day 733. After consultation with her neurologist, amitriptyline at 10 mg was reintroduced, and aripiprazole was discontinued on day 755. Subsequently, BMS symptoms gradually improved without adverse effects on her DLB condition (MMSE: 18/30, HDSR: 14/30). She marked high VAS scores (85/100) but noted that the burning pain manageable provided she avoided certain foods that irritated her tongue. This improvement was accompanied by a lower PCS score (22/52) by day 818, indicating a good prognosis of BMS without severe exacerbation of DLB.

## 3 Discussion

This case report is the first to document a BMS patient who developed DLB during BMS treatment. Notably, the BMS prognosis was favorable, with DLB progressing mildly.

In the initial stages of dementia, differentiating it from mood disorders is difficult since depression and anxiety often coexists (8, 9). The patients with BMS also have higher risks for depression and anxiety, although no significant increase in dementia risk has been reported in comparison to healthy subjects (10). In the sole case report describing BMS as pseudo-psychiatric symptoms (11), the patient was initially diagnosed with BMS but gradually showed delusional sensations and depressive complaints. By the time DLB was diagnosed, the disease had advanced to a stage where treatment was ineffective. The authors highlighted the complex symptom overlapping between BMS and DLB, which posed challenges in diagnosis and treatment. In the present case, symptoms such as anxiety, uncontrolled emotions, and unstable burning sensations could retrospectively be seen as signs of DLB. However, her underlying predisposition to high anxiety, excessively pessimistic psychological characteristics, and coupled with her husband’s physical condition, initially obscured the DLB diagnosis. What ultimately facilitated her prompt referral was the detailed reports provided by her husband regarding the changes in her daily behavior that only he had noticed. As a result, she received appropriate treatment for DLB in the early stages. Visual hallucination is one of the main symptoms of DLB and more found in females than in males (8). Capgras syndrome is a visual hallucination where the patent misidentifies close family members to someone else, is more easily detected by someone really close to patient, as was with the husband in the present case. While significant parkinsonism was not observed in this case, decrease of daily activity level caused by her lumbar compression fracture might have masked movement disorder such as gait disturbance. This case emphasized the reaffirmed necessity of careful interviews regarding changes in daily life, not only with the patients but also with their families, during the medical interview.

Moreover, two main features of DLB, visual hallucinations and parkinsonism are related to dopaminergic system. Aripiprazole which is a dopamine partial agonist might have a negative impact on DLB although it was not evident in the present case. Conversely, anticholinergic medications, commonly used as a first-line treatment for BMS, have been associated with cognitive decline (12). Both BMS and DLB can hardly be improved completely since pathophysiology and effective medications are interact in a complex manner. In the present case, treatments were challenging. Decreasing amitriptyline and switching to mirtazapine, while considering the potential effects on cognitive decline, led to a slight exacerbation of BMS, and discontinuing mirtazapine resulted in sleep disturbances. Maintaining a low dose of amitriptyline consequently had kept BMS stable without severe adverse events, in close collaboration with her neurologist. In addition, rivastigmine, which is a medication for dementia inhibiting acetylcholinesterase and butyrylcholinesterase in the central nervous system, may have had a beneficial effect on both DLB and BMS. Aging induces increase of cholinesterase enzyme which lead to acetylcholine degrading followed by pain exacerbation (13). However, acetylcholine esterase inhibitors including rivastigmine inhibit cholinesterase and activate acetylcholine action by increasing acetylcholine in the synaptic cleft. While amitriptyline activates descending pain inhibitory pathway, rivastigmine also effects on the pain control system (13). These mechanisms may have synergistically contributed to favorable prognosis of BMS without sever exacerbation of DLB in the present case.

In conclusion, the possibility of cognitive decline underlying or induced as adverse events should be considered during the treatment of BMS, especially in the elderly patients, despite the unclear interaction between BMS and DLB. Moreover, a prudent approach to diagnosis and collaboration with specialists are important in managing BMS with the early phase of DLB for improving the prognosis.

## Data availability statement

The original contributions presented in the study are included in the article, further inquiries can be directed to the corresponding author.

## Ethics statement

The studies involving humans were approved by the Ethical Committee of Tokyo Medical and Dental University Hospital. The studies were conducted in accordance with the local legislation and institutional requirements. The patient provided her written informed consent to participate in this case report. Written informed consent was obtained from the individual for the publication of any potentially identifiable images or data included in this article.

## Author contributions

MW: Writing – original draft, Writing – review & editing, Data curation, Investigation. WA: Conceptualization, Data curation, Investigation, Methodology, Supervision, Validation, Writing – review & editing. CT: Data curation, Investigation, Writing – review & editing. CM: Data curation, Investigation, Writing – review & editing. RT: Data curation, Investigation, Writing – review & editing. YK: Data curation, Investigation, Writing – review & editing. GN: Writing – review & editing, Data curation. TT: Investigation, Writing – review & editing. TA: Investigation, Writing – review & editing, Supervision. AT: Data curation, Funding acquisition, Investigation, Methodology, Project administration, Resources, Supervision, Writing – review & editing.
